# Hospital Statistics

**Published:** 1913-11-01

**Authors:** 


### HOSPITAL STATISTICS.

In another part of this issue will be found the second of two articles upon hospital records and statistics as they at present exist in the voluntary hospitals of this country. (The first article was published on October 18, 1913, p. 63.) The criticisms which our contributor offers deserve especial attention because, not content with pointing out defects which many others have complained of, he puts forward a constructive scheme for removing the reproach which at present attaches to the register-keeping in practically all our voluntary institutions. The root of the matter, as he points out, lies in the facts that the officers responsible for the care of the case registers are not trained statisticians, nor in the great majority of cases do they take any intelligent interest in statistics; that they hold their posts for comparatively short periods; and that they deliberately accept such posts as stepping-stones to appointments on the visiting staff, and devote their best energies while they hold them to the acquisition of clinical experience as understudies to the visiting staff. For this state of things the young physicians and surgeons who pass through the registrarships at large hospitals—and only large hospitals as a rule have registrars at all—are not to be blamed. The system is not of their making, and they take it as they find it.

The remedies which our contributor proposes are adapted to the peculiar financial difficulties of most voluntary hospitals; they are not the best he could devise, but the best that he considers practicable. They are, moreover, suitable only to London and such large towns as possess several separate large hospitals. It is not the intention to recapitulate them here, for they are very fully and ably expounded in the article referred to. In directing attention to them, we do so because we are sure that the argument in favour of them is based upon a sound premiss—namely, the hopeless futility of hospital case registers as they are at present kept.

So much is this the fact that everyone who has spent much time in a hospital looks with great suspicion upon any clinical argument founded upon a series of hospital cases. The explanation of this is furnished by the writer of the article, but there is an additional reason which may be mentioned here.

The medical or surgical registrar of a hospital is almost always waiting for a chance of promotion to the staff. Such promotion depends mainly upon the good opinion of the members already on that staff. He cannot therefore afford to make any enemies among them, and indeed it pays him to cultivate their good will. Hence, if any of them requests him from time to time to furnish from among the hospital records figures showing the effects of any special line of treatment which that particular member of the staff has employed, the registrar has a powerful motive for taking optimistic views; and statistics, as is well known, can be made to prove anything. This is, perhaps, an even weaker spot in the system than those upon which our contributor has placed his finger; and of this stupid system reform is distinctly overdue. We do not insinuate that deliberate "cooking" of statistics takes place; indeed we should be the first to repudiate such a charge, if it were made. But the raw materials of hospital statistics—namely, the case registers—are so fundamentally unreliable and confused that no amateur statistician can be trusted to analyse them; and even professionals find the task difficult or impossible to carry out satisfactorily.

Our contributor also touches on the fact that the interpretation of statistics is itself a highly expert matter, and one for which both a special aptitude and a special training are required. Thus not only must the personal factor, as far as possible, be eliminated, which points at least to the same person doing the work for long rather than short periods, as at present, but also a corrective factor is required, such as a central bureau, to which different institutions would send their respective returns, should give. It is easier to state the needed reforms than to embody them in practice. But such a statement is an essential preliminary.

